# Redesigning a Healthcare Demand Questionnaire for National Population Survey: Experience of a Developing Country

**DOI:** 10.3390/ijerph18094435

**Published:** 2021-04-22

**Authors:** Diane Woei Quan Chong, Suhana Jawahir, Ee Hong Tan, Sondi Sararaks

**Affiliations:** Institute for Health Systems Research, National Institutes of Health, Ministry of Health Malaysia, Shah Alam 40170, Selangor, Malaysia; chong.dwq@moh.gov.my (D.W.Q.C.); jdreehong@moh.gov.my (E.H.T.); sararaks.s@moh.gov.my (S.S.)

**Keywords:** health survey, population health, utilization, stakeholder engagement, methodology

## Abstract

Since its inception in 1986, the contents of the National Health and Morbidity Survey (NHMS) have been periodically updated to support emerging health data needs for evidence-based policy and program development. In 2018, the healthcare demand questionnaire was redesigned to capture diverse and changing population demand for healthcare services and their utilization pattern. This paper describes the methods and processes undertaken in redesigning the questionnaire. We aim to highlight the systematic and inclusive approach, enabling all potential evidence users to be involved, indirectly encouraging research evidence uptake for policy and program planning. We applied a systematic approach of comprehensive literature search for national-level population survey instruments implemented globally and translated non-English tools to English. The development phases were iterative, conducted in parallel with active stakeholder engagements. Here, we detailed the processes in the planning and exploratory phase as well as a qualitative assessment of the questionnaire. We included instruments from 45 countries. The majority were from the Organisation for Economic Co-operation and Development (OECD) countries and focused on perceived health, health-related behavior, and healthcare use. Thirty-four stakeholders from 14 areas of expertise were involved. Stakeholders identified additional content areas required, such as chronic pain, alternative use of healthcare services (community pharmacy, home-visit, and private medical laboratory), family doctor, and informal caregiving. The questionnaire, redesigned based on existing literature with concordant involvement and iterative feedback from stakeholders, improved the choice of health topics through the identification of new topics and modification of existing questions to better meet future evidence needs on health problems, strategy, and program planning towards strengthening the nation’s health systems.

## 1. Introduction

Health surveys are essential to gather information on population status that is not routinely collected through statistical or health records. In Malaysia, the National Health and Morbidity Survey (NHMS), a major data collection program for the Ministry of Health Malaysia (MOH), is the principal source of information on the population’s health-related practices. The survey’s main objective is to monitor the health of the people in Malaysia through the collection and analysis of a broad range of health topics, including health status, health-seeking behavior, healthcare utilization, health promotion, and disease prevention [1,2]. Information from this survey is used by policymakers, program managers, and the public health research community to plan for health systems, characterize populations with health problems, determine barriers to access and the use of appropriate healthcare.

The major strength of this population-based survey is that it generates nationally representative data of non-institutionalized population and is able to categorize the population’s health characteristics by demographic and socioeconomic indicators. The survey was first initiated in 1986 and was subsequently carried out in 1996 and 2006 for evidence on healthcare demand and utilization. Since 2011, it was conducted on a four-yearly cycle to provide timely evidence on trends and changes in healthcare demand for health programs’ planning. This survey consists of demographic and socioeconomic information and a rotating core of predetermined topics based on national health priorities [2]. Despite the latest addition of the community’s perception towards healthcare services in 2015 [3], there is a need for the areas covered by the healthcare demand questionnaire [4] to be revisited. It enables policymakers to keep abreast of the current epidemiological transition and trends in utilization, as these are valuable health information for policy development and program planning [5]. It is another agenda for review, given the need for a healthcare system reform and the need for a shift in the delivery of healthcare services from healthcare facilities back to the community and home [6,7]. Additionally, the challenge of demographic transition, particularly the rapid growth of the elderly population in Malaysia [8], and the implementation of pain as the 5th vital sign [9], also contributed to the need to redesign the questionnaire. 

The demand for health and healthcare will continue to increase and evolve [10]. In order to enable the identification of potential areas for improvement, international comparisons with other national-level surveys are essential. Similarities and differences in measuring demand for healthcare across countries create opportunities to learn from other countries. In turn, it could produce comparable, comprehensive, and concise health measures or surveys [11].

During the development of a tool, particularly for a national health survey, engagement with an expanded list of stakeholders with a vested interest in the area, including policymakers, program planners, and healthcare professionals, throughout the process is essential. Besides identifying additional areas to be included in the questionnaire, other potential advantages include maximizing its social benefits, improving policy relevance, and ensuring the questionnaire aligns with its health research priority areas [12]. A study conducted in Canada reported processes involved in developing a new sedentary behavior module for a national health survey. However, engagement with the potential stakeholders is lacking in the study [13]. Another published paper on the development of a health survey to monitor physical activity in the European Union only emphasized the development of the domain for the physical activity questionnaire [14].

In planning for NHMS 2019, in 2017, we initiated the redesign of the healthcare demand questionnaire to improve the relevance of health topics covered and to meet the needs of various stakeholders better. Besides capturing the diversity and changing population dynamics, the goal of the questionnaire redesign was to develop a relevant, clear, and succinct questionnaire [1]. 

Quality testing through iterative pretest and pilot test was an important step in developing the questionnaire [15,16] to ensure respondents’ understanding, clarity of terms used, and relevance of questionnaire items and response options. It, in return, will reduce participant burden, improve response rates, and improve data quality [15,17]. Besides, ensuring a smooth, logical flow of the questions is also crucial as random ordering questions will result in incomplete surveys [18]. The drawback of the method included the fact that the processes are very exhaustive and time-consuming [19].

To the best of our knowledge, to date, limited published studies emphasize the need to involve and engage stakeholders in the development of national health surveys [20]. Documenting the process used in the redesign of this questionnaire would be of value to other researchers. This article summarizes the approaches applied to create an instrument for measuring population health and healthcare demand. It focused on methods and strategies used in the questionnaire development process and could guide future developers of national health instruments.

## 2. Materials and Methods

**Figure 1:**
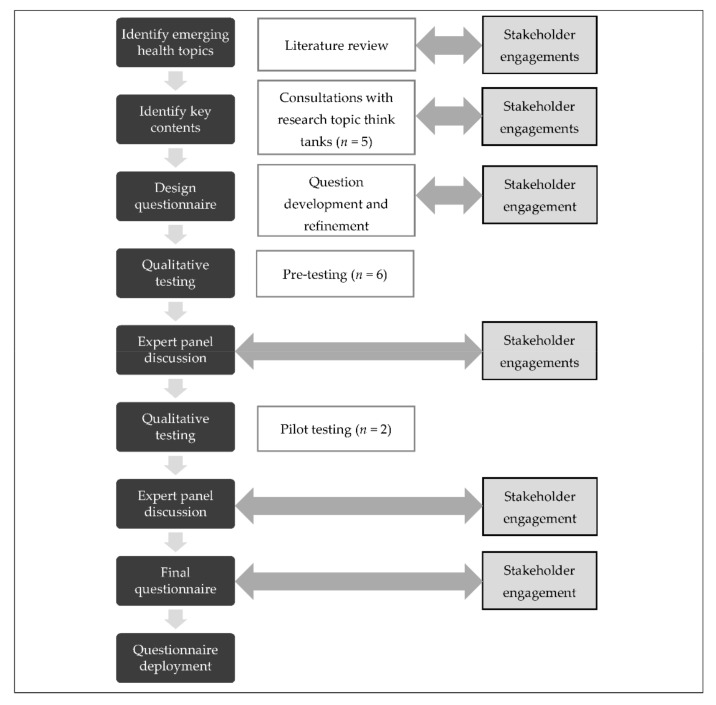
The process adopted for redesigning the questionnaire for the national health survey.

The survey instrument was redesigned from May 2017 to December 2018 in phases. It was refined according to steps documented in the literature [13,15,21]. We included a planning and exploratory phase, followed by a development and quality evaluation phase (Figure 1).

### 2.1. Principles of Redesigning the Healthcare Demand Questionnaire

We applied a systematic approach with a key design feature that incorporates ongoing active engagement with stakeholders throughout the process. The steps conducted are as follows: (1) literature review, (2) consultation with research topic think tanks, (3) development and refinement of questions, (4) pretest, (5) expert panel discussion, and (5) pilot test. 

### 2.2. Phase 1: Literature Review

We conducted an initial and ongoing review of the literature throughout this study. We performed literature searches with PubMed, Web of Science, and Google Scholar databases, as well as Google search engine to identify potential areas that could be adapted from national and international household, health, and general population surveys. Additionally, government websites of selected countries were also searched to ensure comprehensiveness. For survey instruments documented but with no questionnaire available even with supplemental web-based search, we emailed the authors/survey implementers to request for the instrument. We reviewed all instruments to identify relevant research areas and questions.

Following this, we developed an inventory of questions based on Andersen’s Behavioural Model of Health Services Use [22], with items thematically mapped to facilitate stakeholder and expert panel discussions using Microsoft Excel. This map of existing survey tools and items, coded, informed the scope of early drafts of the questionnaire. This work was further explored through a series of iterative engagement with stakeholders. 

### 2.3. Phase 2: Consultation with Research Topic Think Tanks

We consulted multidisciplinary stakeholders intending to gather expert opinions and diverse disciplinary perspectives to inform the questionnaire redesign. Stakeholder selection was based on expertise and included policymakers, program planners, and healthcare professionals for a broad and heterogenous view on current and emerging healthcare trends.

We convened five discussion sessions. Each session began with background presentations and an explanation of goals as well as session objectives. The input was solicited from each stakeholder with facilitated discussions based on session objectives, such as focusing on additional research areas to be incorporated into the national survey tool. Findings from previous NHMS and literature reviews were also shared. Additional literature was retrieved and reviewed on the spot to inform the discussion, and further searches were performed in preparation for subsequent discussion sessions. All stakeholders participated in the discussion and provided pragmatic feedback in identifying items to retain as well as crucial research areas not previously covered. Notes were taken to ensure thorough documentation for questionnaire refinement. An extensive set of research topics was shortlisted for conceptualization and questionnaire construction based on collective stakeholders’ feedback.

### 2.4. Phase 3: Question Development and Refinement

Based on the literature review and input gathered from stakeholder engagements, we conceptualized and constructed questions by creating new questions or adapting items from other national surveys. The survey items were drafted to represent the construct of interest using simple language. We followed a set of criteria for questionnaire development and avoided double-barrel and negatively worded items [21].

The questions were drafted in English and subsequently translated to the Malay language by researchers who were well-versed in both languages. These two languages were used as they were the most spoken languages among the population in Malaysia. These questions were then merged with the questions that were retained. All researchers reviewed the bilingual version of the questionnaire and reconciled discrepancies. The draft questionnaire was circulated amongst stakeholders for additional feedback to ensure that the construct’s conceptualization made theoretical sense to the stakeholders before evaluating the instrument.

### 2.5. Phase 4: Qualitative Testing—Pretest

We conducted practical training for six research members before pretesting the questionnaire with volunteers from the general public. It was conducted among staff-members in two separate groups with varied educational backgrounds, mainly to ensure that the questions were put into meaningful order and format, including question flow and structure of opening questions. This is crucial as the random ordering of questions will result in incomplete surveys [18].

We then conducted six rounds of pretests at 10 public primary health clinics in selected locations, using purposive sampling for appropriate representation of participants from various subgroups based on age, language, and ethnicity. Individuals residing in Malaysia in the past two weeks before the qualitative testing period were included in the testing (pretests and pilot tests). We excluded those who refused to participate. The sites chosen represent diverse locality in Malaysia, namely rural, suburban, and urban. This diverse selection is crucial to ensure that the questionnaire is suited for the general population in Malaysia, and participants interpreted the items in the manner the survey intended. The survey’s actual implementation will be conducted using a tablet to facilitate face-to-face assisted interviews in a representative sample of the population [4].

A cognitive debriefing session immediately followed each pretest. Verbal probing was conducted to gather the participants’ understanding of the terms, clarity of words, questionnaire items’ relevance, and response options [15,21,23]. The targeted sample size for cognitive debriefing is 10 interviews per cycle [24] and it was conducted among adults aged 18 years and above. Quality evaluation of the questionnaire was conducted using an iterative approach whereby in parallel with the series of pretests, revision and refining were conducted on successive questionnaire drafts.

### 2.6. Phase 5: Expert Panel Discussion

As part of active stakeholder engagement, we convened three sessions of expert panel discussions consisting of multidisciplinary stakeholders (clinical consultants, policymakers, public health experts, and survey methodologists in the areas to be addressed) for a detailed review of the questionnaire items. This was convened prior to and after the pilot test. During the session, the items were scrutinized based on the importance of information for the strategic planning of health services and the feasibility of obtaining information through a nationwide survey. Panel members were also allowed to provide written feedback based on their area of expertise. Additional modifications were made based on this input before the final stages of questionnaire refinement.

### 2.7. Phase 6: Qualitative Testing—Pilot Test

We conducted two rounds of pilot tests for the questionnaire among people who attended the selected healthcare facilities and households in chosen states. Participants were selected using purposive sampling. We made special arrangements with healthcare personnel providing home-visit nursing services, mainly targeting the population receiving home-visits and providing informal care to test the related questions [15,18].

Issues encountered and feedback received during the qualitative testing were documented and summarized. Actions for each issue that arose during the pretests and pilot tests were resolved in discussions to achieve consensus among team members. We employed several strategies to overcome these challenges, including modifying the questions, refining the wordings, adding instructions, and preparing a manual for data collection and scenarios for prospective research assistants training during the actual implementation of the survey in 2019. Feedback from the participants was used to further iron out the problems encountered and refine the questionnaire.

### 2.8. Phase 7: Final Questionnaire

We emailed the final questionnaire to all stakeholders to keep them up to date with what has changed and communicate how their feedback is helpful and relevant. They also were informed of what would happen next, allowing them to keep track of its progress.

### 2.9. Phase 8: Questionnaire Deployment

The final questionnaire was shared with the national survey implementers in September 2018 to develop the survey systems. The questionnaire was then used to collect data for NHMS 2019, between July and October 2019 [4].

### 2.10. Ethical Considerations

This study was registered in the National Medical Research Registry (NMRR), bearing registration number NMRR-17-905-35933. All participants provided written informed consent to participate in the study. Anonymity and confidentiality of participants in this study were assured, with no personal identifiers collected.

### 2.11. Analysis

The demographic and socioeconomic characteristics of the participants were analyzed using descriptive statistics. Qualitative analysis of findings from the pretest and pilot test using cognitive debriefing was conducted [17].

## 3. Results

### 3.1. Literature Review

**Figure 2:**
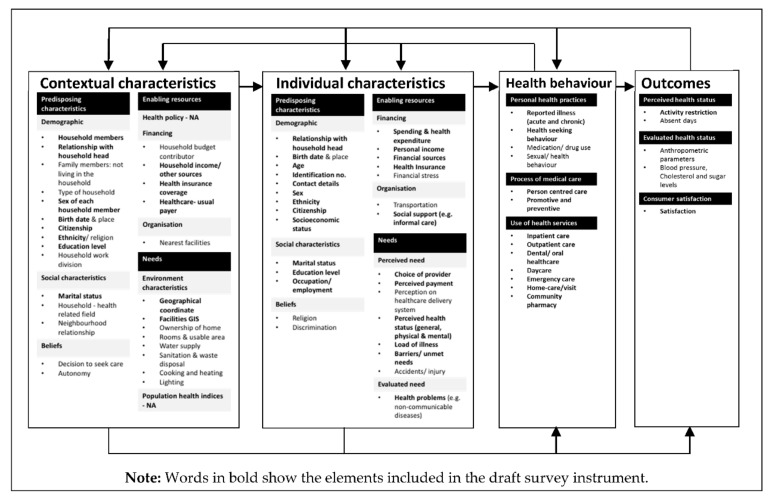
Domains mapping based on Andersen’s Behavioural Model of Health Services Use.

Overall, we gathered references from 48 countries. However, instruments from three countries were excluded in this study as we were unable to translate them. Instruments from 45 counties were included in the development of the questionnaire, of which the majority were from the Organisation for Economic Co-operation and Development (OECD) countries. Results of the domain mapping from all the references are shown in Figure 2. The majority focused on perceived health, health-related behavior, and the use of healthcare. Moreover, these surveys also cover health topics such as disease-specific morbidity, chronic conditions, use of medicines, and others.

Overall, almost all demographic and social characteristics for both contextual and individual characteristics were incorporated in the draft survey instrument, including birth date, age, sex, ethnicity, citizenship, socioeconomic status, marital status, and education level. For health behavior, all types of utilization of health services were incorporated in the draft survey instrument, following views and feedback received from the expert panels and stakeholders.

### 3.2. Stakeholders Engaged

Altogether, 34 stakeholders covering 14 areas of expertise were involved in the development process of the questionnaire. Table 1 listed panel members’ expertise comprising policymakers and clinical experts from the public sector engaged in the process of redesigning the questionnaire.

### 3.3. Content Areas Added and Dropped

There were several main topics included in the healthcare demand questionnaire with different target populations since 2011. The main topics covered were household, sociodemographic and socioeconomic, payer for healthcare, general illness, utilization of outpatient healthcare, utilization of inpatient healthcare, and utilization of oral healthcare. In NHMS 2019, the additional content areas identified by the expert panels and stakeholders included general health, chronic pain, utilization of healthcare services (community pharmacy, home-visit, and private medical laboratory), family doctor, and informal caregiving. Previously, general health was included in 2011, and chronic pain was covered in 2006. However, they were dropped in the following cycles, and both were included back in NHMS 2019 as they were deemed necessary by the stakeholders. The exact questions from the earlier cycle that were added to this cycle were reincorporated with only slight amendments to improve the clarity of the questions. The meaning of the questions remained the same, and this was confirmed through review sessions with the stakeholders. This ensured that the questions in the added module were comparable in both cycles. The community’s perception towards healthcare services, which was covered in 2015, was dropped in 2019 as suggested by the stakeholders. There is no requirement to gather additional evidence for this in a short period.

### 3.4. Survey Respondents

In total, 242 and 113 respondents participated in the pretests and pilot tests, respectively. During pretests, most of the respondents were female (59.3%), Malay (63.2%), Malaysian (94.0%), married (73.1%), completed secondary school (48,.4%), and employed (62.1%). For pilot tests, half of the respondents were female (54.8%), aged between 50 and 69 years old (54.8%), completed secondary school (50.0%), and unemployed (59.5%). Findings from the pretest and pilot tests are summarized in tables as we refined and improved the questionnaire’s quality. Table 2 summarizes the characteristics of the respondents during qualitative testing.

Table 3 presents the examples of action taken for issues that arose during the pretests and pilot tests. Particular attention was also paid to the position, flow, and recall period of the questions. Since the healthcare demand questionnaire covered several topics, with varied recall periods, the questions on the utilization of health services were positioned right after questions on general health and load of illness to trigger respondents’ memory on the health condition that might lead to health services use. This prompting effect could reduce the probability of underreporting due to poor recall. We retained the recall period for outpatient care and inpatient care utilization to ensure healthcare utilization estimates’ consistency. Meanwhile, the recall period for the new topics was determined based on the literature review and expert opinions.

The final healthcare demand questionnaire comprised 10 topics: (1) household, (2) sociodemographic and socioeconomic, (3) payer for healthcare, (4) general health and illness, (5) utilization of community pharmacy, (6) utilization of outpatient healthcare, (7) utilization of inpatient healthcare, (8) utilization of oral healthcare, (9) home-visit, and (10) informal care [4].

## 4. Discussion

National Health and Morbidity Survey is the principal source of information on the health and demand for care of the country’s non-institutionalized population, with study findings used to inform healthcare planning since its inception in 1986. To provide timely and relevant supporting data to support the health systems in addressing health issues and anticipating emerging healthcare challenges, the use of multiple components in an iterative process in redesigning the questionnaire proves extremely useful to update and revise contents to suit health systems planning needs. In September 2018, we shared the final questionnaire with the national survey team responsible for developing information systems for data collection. The questionnaire has been used for National Health and Morbidity Survey (NHMS) 2019, which has been completed successfully in August 2020 [4,5,25]. The questionnaire is available for download here: http://www.ihsr.moh.gov.my/images/publication_material/NHMS2019/hcd2019_report.pdf (accessed on 22 February 2021). This paper aims to describe the approaches applied to develop an instrument to measure population health and healthcare demand, step-by-step. 

Survey questions must be based on the best available evidence, be valid and reliable [2,13]. In NHMS, the contents must be feasible to be measured through a community survey, were inaccessible via a routine monitoring system, and more appropriately collected through a community survey. It addresses crucial priorities for the nation, and the prevalence is high for estimating sample size [26]. The questionnaire has to be concise, not too long, and understandable for a multiethnic population to ensure quality responses [27].

The systematic evaluation of questionnaire items, decision-making with iterative stakeholder engagement, as well as the thorough and inclusive discussion on the rationale for items to be added, deleted, retained, and modified was beneficial. An in-depth description in preparation for the population-based national survey and a developing country’s experience on how to redesign a population survey questionnaire could enable researchers and policymakers to understand the contents and resultant findings better.

In redesigning our historical questionnaire, the key design feature was ongoing, active engagement with stakeholders. The initial engagement with stakeholders included technical expert consultation sessions to identify additional content required. This proved to be an enriching experience for researchers to understand health decision-making and program planning better. Prior to the discussion, the participants were provided with necessary information on the research background and the mapping of available survey tools, as well as the goals and objectives for the engagement. This pre-empting of stakeholders was an important step before setting the stage and achieving meaningful contributions [12]. Stakeholder engagement opportunities can be at any particular point of research steps or throughout the whole research process, from formulating the initial research question to disseminating findings. The engagement is integral for identifying and prioritizing topics to generate evidence, which is relevant and useful to potential knowledge users, increase dissemination and uptake of research findings to support and inform healthcare decisions [28]. 

Catering to the stakeholders’ needs, we reintroduced questions related to chronic pain in the final questionnaire to measure the prevalence of chronic pain sufferers among the Malaysian population and the effect of the pain on their daily activities work. In Malaysia, since the implementation of the “Pain as the 5th Vital Sign” initiative in 2008 and the “Malaysian Pain-Free Hospital (PFH)” initiative in 2011 [29], there was an absence of national data on the prevalence of chronic pain sufferers among the population. The findings from the NHMS 2019 survey will help in the planning of suitable interventions related to pain management for the population in Malaysia.

Besides, information on how people perceived their health status was an important factor for the survey’s inclusion. It offers a comprehensive picture of their physical and emotional well-being, the ability to predict health-seeking behavior, and the use of healthcare in the population. Although subjective in nature, self-reported health is a strong indicator of potential health care demands and mortality [30,31]. As the current study found that the question on how people perceived their health, in general, was included in almost half of the total number of countries reviewed, it was incorporated in the national survey to allow comparison with other countries.

Meanwhile, the growing elderly population is anticipated to affect the demand for all healthcare services. It is expected that the percentage of older persons aged 65 years old and over in Malaysia will increase from 5% in 2010 to 14.5% in 2040 [8]. The aging population places pressure on health systems by growing the need for care to prevent and treat noncommunicable diseases and chronic conditions associated with the elderly [32]. However, the challenges can be overcome by anticipating future demographic changes and enacting policies to respond proactively to the aging population. Therefore, the final questionnaire included topics on home-visit and informal care. This is to measure the demand for care, particularly among the aging population. The related topics will give policymakers valuable information to table policies that aim for better mechanisms to meet the ever-growing need for care, especially among the older population.

Qualitative evaluation of the instrument was attained through a series of pretest and pilot tests conducted in parallel with the questionnaire’s refinement. This enabled the researchers to gather valuable insight from prospective participants, for example, on the terms’ understandability, comprehensibility of the new questions, acceptability of the structure, and format of the questionnaire, and response options. Items with vague concepts could be readily identified during the qualitative testing and rephrased to best suit the population, given Malaysia’s diversity of languages and cultures. This is crucial as the sample must represent diverse populations with people from different age groups, socioeconomic status, races, or ethnicities. The questionnaire will be fielded in a multicultural and multiracial setting [16]. Furthermore, testing done among individuals and households for each household member was crucial, in line with the standard criteria to test the instrument in the same data collection mode as the final survey [17,21]. By conducting six rounds of cognitive interviews with iterative item refinement between each round, we arrived at a bilingual (Malay–English) version of the questionnaire that was understandable to the majority of cognitive interviewees.

As our questionnaire recorded responses in descriptive forms and does not involve the scaling method, we only conducted a qualitative analysis of the instrument throughout this study. We highlighted the qualitative analysis of the results to suggest valid and effective strategies in detecting problems that may lead to an error in the survey response. The pretest is considered to be an empiric assessment of the questionnaire in which we performed cognitive debriefing in this study. However, for a survey questionnaire that uses a scaling method to generate a health index or measure a construct, quantitative analysis to demonstrate the validity and reliability is necessary to ensure the questionnaire’s usefulness [33].

There is a challenge in an ongoing survey between keeping updated and maintaining continuity. We tried our best to keep the main topics, such as outpatient healthcare, inpatient healthcare, and oral healthcare, consistent so that trends could be tracked through the NHMS [34,35], and the remaining topics were revised in response to stakeholders’ requests. The amount of time it took respondents to complete the questionnaire was also taken into account, as the longer questionnaire contributes to the burden on respondents [27]. The questionnaire’s scope and length were allowed for NHMS 2019 based on the estimated average time to complete the questionnaire being held at 45 min to ensure quality data. A consensus was reached among the stakeholders, researchers, and survey implementors on the survey scope and length prior to the questionnaire deployment.

### Strengths and Limitations of this Study

Issues or item problems raised during the qualitative testing were consolidated. The most appropriate refinement strategy of either adding, deleting, retaining or modifying the questions prior to the next field-testing was chosen through group-discussion among the research team and relevant stakeholders. This proved beneficial to the questionnaire redesign [36]. Furthermore, purposive sampling during the qualitative testing yielded participants of various demographic and socioeconomic backgrounds, enabled testing in both languages of the questionnaire, and allowed testing of uncommon topics.

Despite these key strengths, there were several limitations in our study. Firstly, our literature search focused on instruments used in population-based national surveys on demand and utilization of healthcare. We may have missed out on survey instruments that were implemented in smaller settings. Secondly, we did not include groups of stakeholders from academics, civil groups, and lay citizens during the exploration phase of additional research areas to be incorporated into the survey. Instead, we gathered feedback from lay citizens and healthcare users during the iterative rounds of qualitative testing. Thirdly, we could not conduct quality evaluation until we reached saturation for each cycle of pretest and pilot test and may have missed some problematic issues with the survey items [37,38], although this could be detected at the quality evaluation stage.

## 5. Conclusions

The questionnaire was redesigned based on existing literature with concordant involvement and iterative feedback from stakeholders. Based on Andersen’s Behavioural Model of Health Services Use, the survey instrument included almost all demographic and social factors to identify contextual and individual characteristics, including birth date, age, sex, ethnicity, citizenship, socioeconomic status, marital status, and level of education. The questionnaire was developed with the participation of 34 stakeholders from 14 different fields of expertise. All forms of health service use were included in the draft survey instrument following the stakeholder engagement sessions. General health, chronic pain, utilization of healthcare services (community pharmacy, home-visit, and private medical laboratory), family doctor, and informal caregiving were among the additional subject areas identified by the expert committees and stakeholders in NHMS 2019. Identification of new topics and modification of existing questions had improved the choice of health topics covered in the survey. Quality testing through pretest and pilot test was an essential step in developing the questionnaire to reduce participant burden, improve response rates and data quality.

## Figures and Tables

**Table 1:** Stakeholders involved in the questionnaire redesign process, based on the area of expertise (*N* = 34).

Area of Expertise (n)	
Health policy (4)	Pharmaceutical care (2)
Family health (2)	Traditional and complementary medicine (2)
Public health (3)	Geriatric care (1)
Oral health (1)	Oncology (1)
Health planning (4)	Pain condition and management (1)
Health financing (8)	Palliative care (1)
Health statistics (2)	Questionnaire design and survey methodology (2)

**Table 2:** Characteristics of respondents during qualitative testing.

Characteristic	Pretest (*N* = 242) Number of Cycles: 6		Pilot Test (*N* = 113) Number of Cycles: 2	
	n	%	n	%
**Sex**				
Male	87	36.0	51	45.1
Female	151	62.4	62	54.9
**Age (years)**				
17 and below	6	2.5	2	1.8
18–29	44	18.2	18	15.9
30–49	90	37.2	26	23.0
50–69	84	34.7	56	49.6
70+	14	5.8	11	9.7
**Ethnicity**				
Malay	161	66.5	74	65.5
Chinese	25	10.3	13	11.5
Indian	43	17.8	25	22.1
Others	7	2.9	1	0.9
**Citizenship ^1^**				
Malaysian	201	94.8	113	100.0
Non-Malaysian	5	2.4	0	-
**Marital status ^1^**				
Never married	33	15.6	20	17.7
Married	157	74.1	79	69.9
Separated/Divorced/Widow(er)	19	9.0	14	12.4
**Education level ^1^**				
No formal education	12	5.7	11	9.7
Completed primary education	22	10.4	13	11.5
Completed secondary education	101	47.6	59	52.2
Completed tertiary education	76	35.9	30	26.6
**Employment status**				
Yes	141	58.3	61	54.0
No	101	41.7	52	46.0

^1^ Total number of respondents for pretests was 212.

**Table 3:** Examples of action taken based on results of the qualitative testing.

Issue	Original Item	Action Taken	Revised Item
Reasons for no coverage of any personal health insurance plans were identified as important to cater to current demand and assist in policy-making.	Are you covered by any personal health insurance plans which you or a family member had purchased?	New question added	Are you covered by any personal health insurance plans which you or a family member had purchased? If no, why?
Information on how people perceived their health generally was identified as important. The literature review found that the question was included in almost half (21) of the total number of countries included in the review.	How would you rate your health?	Question added	-
The question was problematic to measure the prevalence of chronic pain among participants. For instance, one respondent mentioned muscle pain after physical activity, which will eventually go away after rest. Thus, a duration to imply chronic pain and definition of persistent pain was included in the question’s text.	In the last two weeks, from [fill month and year] till today, did you experience any bodily pain? (e.g., headache, joint pain, muscle aches, etc.). If yes, how long you have been experiencing the pain?	Accept original question with major edits	In the last six months, from [fill month and year] till today, have you had persistent pain in any part of your body lasting for three months or more? (Persistent pain means that the pain is felt every day, or most days, during that period).
Cognitive testing confirmed that participants were primarily thinking of pharmacies with a pharmacist who sells medicines when they responded to this question, as intended.	In the last two weeks, from [fill month and year] till today, did you go to a community pharmacy for yourself or other health reasons?	Accept original question	-
Testing revealed that participants have difficulty estimating the duration when they last received dental care. The answer options were simplified into multiple choice answers.	When was the last time you received dental care? (please write estimated month and year) Original answer options: … month … year Never received	Modified	When was the last time you received dental care? (please write estimated month and year) Final answer options: 1–2 years ago More than 2 years ago Never received
Testing revealed that participants have difficulty answering the questions on which person they cared for. For instance, one respondent who had provided care for two persons reported the same amount of time spent for both persons, but one is in the household, and another is not in the household.	If assists more than one, the respondent only has to answer for one individual cared for.	New clearer instruction used in the questionnaire with additional guideline in the manual to guide the interviewers during data collection	Instruction: If assists more than one, the respondent only has to answer for one individual who is most often taken care of. Additional guideline: Adopted from: Survey of Carers in Households—England, 2009–2010. a. If assists more than one person, select the one that the respondent spends most time helping. b. If the same amount of time is spent assisting two people, select the one that lives in the respondent’s household. c. If the same amount of time is spent assisting two people, both of whom live in the respondent’s household, select the person on whom more time is spent. If the respondent is unable to say for which person, she/he spends most time caring, select the first one listed. d. If there is more than one person cared for and they all live outside the household, select the one with the highest number of hours helped. e. If the same amount of assistance is given to more than one person, all of whom live outside the household, choose the first one listed.
The question was problematic as participants tend to answer 24 h per day (168 h per week) when they perceive care provision is intensive, excluding the number of hours when they are asleep. For participants who were not living in the same household with the cared-for person, the participants tend to exclude the traveling time to and from their home.	In total, how many hours per week do you normally spend providing the care to [fill cared-for person’s name]?	Accept original question with minor edits	If the cared-for person is in the household: In total, how many hours per week do you normally spend providing the care to [fill cared-for person’s name], apart from when you are asleep? If the cared-for person is not in the household: In total, how many hours per week do you normally spend providing the care to [fill cared-for person’s name], including time traveling to and from his/her home?
Testing confirmed that the question was not necessary, as it was already mentioned in the instruction that informal care does not involve wage/salary.	Were you paid to provide care?	Question dropped	-
Reasons for no coverage of any personal health insurance plans were identified as important to cater to current demand and assist in policy-making.	Are you covered by any personal health insurance plans which you or a family member had purchased?	New question added	Are you covered by any personal health insurance plans which you or a family member had purchased? If no, why?

## Data Availability

The data presented in this study are available on request from the corresponding author on reasonable request and permission from the Director-General of Health, Malaysia. The data are not publicly available due to protect participant privacy.
